# Latent profile characteristics of nature connectedness and place attachment among urban older adults practicing Tai Chi: psychological heterogeneity of green space exposure

**DOI:** 10.3389/fpubh.2026.1912037

**Published:** 2026-09-17

**Authors:** Xiaoli Huang, Tongtong Hao, Zhihong Huang

**Affiliations:** 1 Jiangsu Normal University KeWen College, Xuzhou, China; 2 College of Chinese Wushu, Beijing Sport University, Beijing, China

**Keywords:** active aging, individual heterogeneity, latent profile analysis, nature connectedness, older adults, place attachment, Tai Chi exercise, urban green space

## Abstract

**Background:**

With the rapid acceleration of population aging, promoting active aging and psychological health among older adults has become an important public health priority. Urban green spaces play a key role in supporting physical activity and psychological adjustment in older adults. However, most existing studies have adopted a variable-centered approach, which limits the understanding of intra-individual psychological heterogeneity. This study focused on urban park older adults practicing Tai Chi and examined latent profile characteristics of nature connectedness and place attachment from a person-centered perspective.

**Methods:**

A cross-sectional survey design was conducted, including 462 urban older adults practicing Tai Chi in Xuzhou, Jiangsu Province, China. Nature connectedness was measured using the Connectedness to Nature Scale across three dimensions (affective, cognitive, and experiential), and place attachment was assessed using the Place Attachment Scale across two dimensions (place dependence and place identity). Latent Profile Analysis (LPA) was used to identify potential subgroups. Chi-square tests and one-way ANOVA were applied to examine differences in sociodemographic and behavioral variables across profiles.

**Results:**

Three latent profiles were identified: High connectedness–High attachment type (C1, *n* = 154, 33.3%), Low connectedness–Low attachment type (C2, *n* = 188, 40.7%), and Functional dependence type (C3, *n* = 120, 26.0%). Significant differences were observed among profiles in age, education level, occupation, and income (*p* < 0.05). In contrast, most behavioral variables (such as Tai Chi practice frequency and years of practice) showed limited differences, except for practice duration. The High connectedness–High attachment type showed the highest scores across all psychological dimensions, while the Low connectedness–Low attachment type showed the lowest.

**Conclusion:**

Urban older adults with regular Tai Chi practice exhibit significant heterogeneity in nature connectedness and place attachment, with differences mainly reflected in psychological rather than behavioral dimensions. Tai Chi practice may play a supportive role in strengthening psychological connections between older adults and urban green spaces. These findings provide evidence for developing tailored health promotion strategies and optimizing urban green space planning based on individual heterogeneity.

## 1 Introduction

With the rapid acceleration of global population aging, promoting physical and mental health as well as active aging among older adults has become a major public health priority (1). In the context of rapid urbanization, older adults’ daily living environments are increasingly shifting toward community spaces and urban parks (2). Urban green spaces are not only important settings for physical activity, but also key environments for psychological regulation and social interaction (3). Therefore, understanding the psychological relationships between older adults and natural environments, as well as activity settings, is essential for explaining the formation of health-related behaviors (4).

In recent years, nature connectedness has emerged as an important concept in environmental psychology and health research (5). It refers to the extent of an individual’s emotional, cognitive, and experiential connection with nature (6). Previous studies have shown that higher levels of nature connectedness are associated with better mental health, lower levels of depression and anxiety, and higher levels of well-being (7). In addition, nature connectedness has been suggested to promote participation in outdoor physical activity, thereby indirectly contributing to improved physical health (8).

Another key environmental psychological construct related to nature connectedness is place attachment (9). Place attachment typically includes two dimensions: place dependence and place identity (10). These reflect the functional reliance on a specific environment and the emotional and identity-based connection to that place, respectively. Research has shown that place attachment not only strengthens individuals’ willingness to continuously use specific environments, but is also closely associated with mental health, social belonging, and life satisfaction (11).

Although previous studies have examined the effects of nature connectedness and place attachment on health-related behaviors, most have adopted a variable-centered approach, treating populations as homogeneous groups (12). However, individuals often differ significantly in their environmental psychological experiences, and distinct subgroups may exhibit different psychological configuration patterns. Such heterogeneity is particularly evident among older adults, as their lifestyles, health conditions, socioeconomic resources, and previous environmental experiences are highly diverse. For example, while some older adults may develop strong emotional connections with natural environments and identify urban green spaces as meaningful places, others may primarily use these spaces for functional purposes such as physical activity or social interaction. These distinct psychological patterns may coexist within the same population and cannot be adequately captured by approaches that estimate only average associations between variables (13).

Tai Chi is a traditional Chinese mind–body exercise that is commonly practiced in urban green spaces such as parks (14). In this study, Tai Chi practice is not treated as an independent predictor or a moderating variable; rather, it serves as a contextual characteristic that defines a population with sustained interaction with urban green spaces (15). The repeated, place-based, and socially embedded nature of Tai Chi practice provides an appropriate context for examining how older adults psychologically connect with natural environments and specific activity settings.

Therefore, urban older adults practicing Tai Chi represent an important population for studying nature connectedness and place attachment. Their psychological experiences in urban green spaces may present complex and heterogeneous structural patterns (Figure 1).

**Figure 1:**
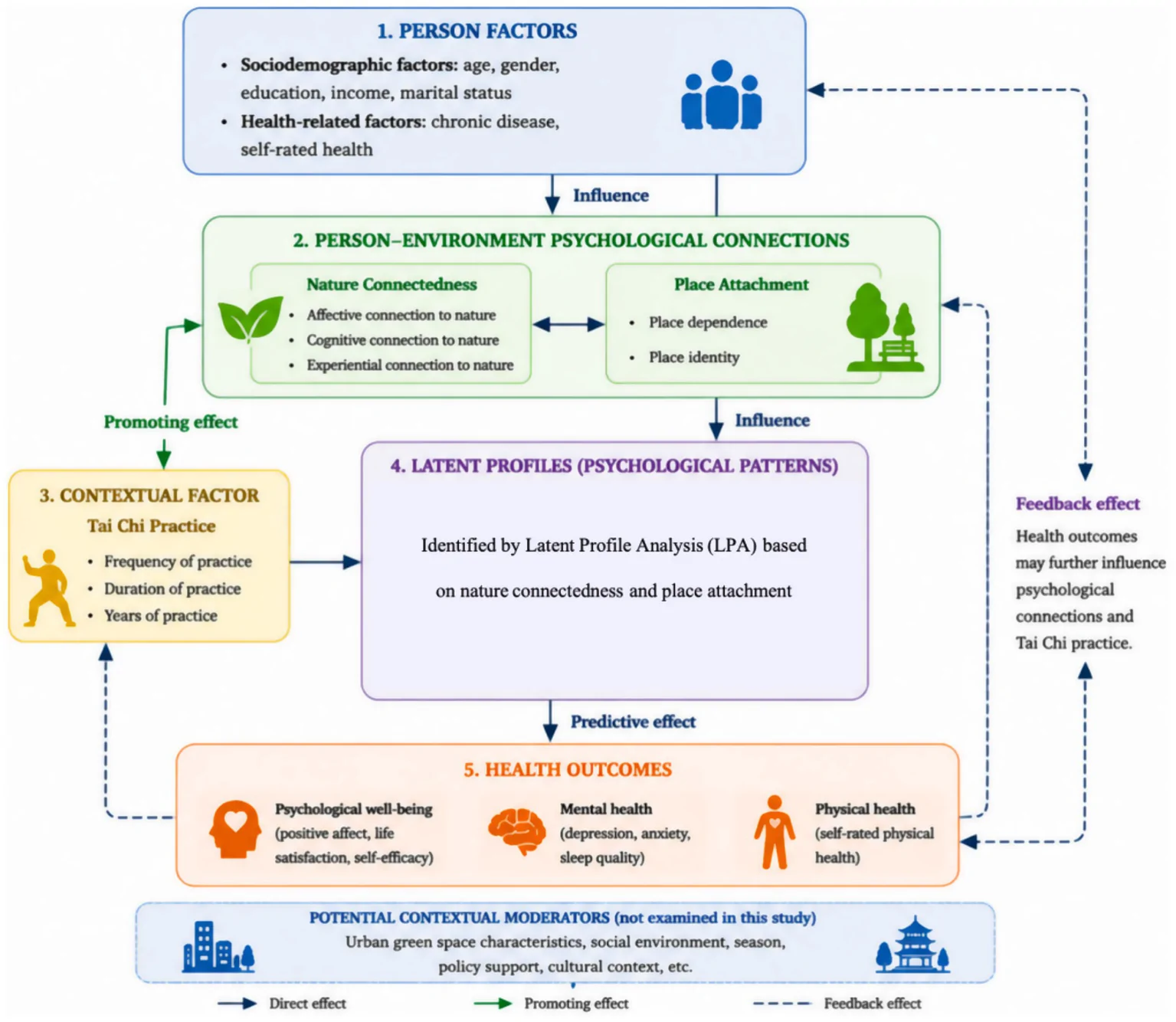
Illustrates the conceptual framework of the relationships among Tai Chi practice, person–environment psychological connections, latent profiles, and health outcomes.

Based on this framework, a person-centered approach was adopted. In particular, Latent Profile Analysis (LPA) was used to identify potential subgroups based on multiple psychological indicators. This approach allows for the identification of unobserved heterogeneity in multidimensional psychological constructs across individuals, rather than assuming population homogeneity. However, empirical evidence on latent classifications of nature connectedness and place attachment among older adults remains limited. In particular, few studies have focused on urban older adults practicing Tai Chi in public green spaces, and systematic examinations of their psychological heterogeneity are still lacking (16).

Therefore, this study focused on urban older adults practicing Tai Chi in urban parks. Using multidimensional indicators of nature connectedness and place attachment, LPA was applied to identify latent profile structures. Furthermore, differences in sociodemographic and behavioral characteristics across profiles were examined. The study aims to deepen the understanding of psychological relationships between older adults and urban green spaces from a perspective of individual heterogeneity. The findings are expected to provide theoretical evidence for precision-based health promotion strategies and the optimization of urban green spaces.

## 2 Methods

### 2.1 Study design and participants

A cross-sectional survey design was adopted in this study to examine the latent profile characteristics of nature connectedness and place attachment among urban older adults practicing Tai Chi in Xuzhou, Jiangsu Province, China (17). The study further aimed to explore differences in sociodemographic and behavioral characteristics across latent profiles (18). The study population consisted of older adults who regularly participated in Tai Chi practice in urban parks. Data were collected through on-site questionnaire surveys conducted in multiple urban parks and public green spaces in Xuzhou, Jiangsu Province. A multistage convenience sampling strategy was adopted. First, based on the classification of urban public spaces in Xuzhou, three types of commonly used exercise environments were considered: comprehensive parks, community parks, and public squares. Parks and squares with relatively high concentrations of older adults practicing Tai Chi were identified through preliminary field observations and consultations with local community fitness organizers. Second, several representative sites from each type of public space were selected according to accessibility, frequency of Tai Chi activities, and willingness of site managers or participants to participate. On-site surveys were conducted during peak Tai Chi practice periods (mainly early morning and late afternoon) on both weekdays and weekends to capture participants with different activity patterns. Third, eligible older adults were recruited using convenience sampling at each selected site. Although probability sampling was not feasible due to the absence of a complete sampling frame of older Tai Chi practitioners, efforts were made to enhance sample diversity by including different types of public spaces and conducting surveys across multiple locations and time periods. After excluding incomplete responses, patterned responses, and clearly invalid questionnaires, 462 valid cases were retained for final analysis (19). All participants volunteered to take part in the study. They were fully informed about the study purpose and procedures before providing written informed consent. This study was approved by the Ethics Committee of Beijing Sport University (Approval No. 2025347H).

Inclusion criteria: (1) Aged ≥60 years and able to independently perform activities of daily living; (2) Regular participation in Tai Chi practice in fixed locations such as urban parks or public green spaces for at least 3 months, ensuring stable behavioral patterns and environmental exposure; (3) Participation in Tai Chi practice at least once per week; (4) Ability to understand the questionnaire and complete it independently; (5) Willingness to participate and provision of written informed consent (20). This criterion was adopted to ensure that participants had sufficient and relatively stable exposure to Tai Chi practice environments, which was essential for examining psychological relationships with urban green spaces.

Exclusion criteria: (1) Diagnosed severe cognitive impairment (e.g., Alzheimer’s disease or severe cognitive dysfunction) or severe mental disorders (e.g., schizophrenia or severe depressive episodes) that may affect comprehension or response validity; (2) Severe physical illness or functional impairment preventing independent completion of the questionnaire; (3) Experience of major life or health events within the past 3 months (e.g., major surgery or acute hospitalization) that may significantly influence psychological status or behavioral patterns; (4) Incomplete questionnaires, patterned responding, or logically inconsistent responses identified during data screening (21).

Among the 462 valid participants, 205 participants (44.4%) were recruited from comprehensive parks, 162 participants (35.1%) were recruited from community parks, and 95 participants (20.6%) were recruited from public squares (Table 1). The distribution of participants across these three types of public spaces reflected the actual availability and frequency of Tai Chi practice among older adults in urban public environments.

**Table 1:** Distribution of participants by type of public space.

Public space type	*n*	%
Comprehensive parks	205	44.4
Community parks	162	35.1
Public squares	95	20.6
Total	462	100

### 2.2 Measures

#### 2.2.1 Sociodemographic and behavioral variables

Sociodemographic and behavioral characteristics of participants were collected, including age, gender, education level, occupational type, and monthly income (22). Age was treated as a continuous variable, while other sociodemographic variables were categorical (23). Tai Chi-related behavioral characteristics were assessed using self-reported questionnaire items. The assessment included practice frequency, practice duration, years of Tai Chi participation, and distance to the practice site. Practice frequency was measured by asking participants the average number of Tai Chi sessions performed per week. Responses were recorded as sessions/week and categorized into three groups according to the distribution of participants: 3–4 times/week, 5 times/week, and 6–7 times/week. Practice duration referred to the average length of each Tai Chi session and was measured in minutes. Participants reported their usual duration of each practice session. In the original questionnaire, practice duration was recorded as a continuous variable rather than being restricted to predefined intervals. For descriptive analysis, duration was categorized into four groups based on the observed distribution: ≤40 min, 41–50 min, 51–60 min, and >60 min. Years of practice were recorded as the number of years since participants began regular Tai Chi participation. Distance to the practice site was measured as the approximate walking distance from home to the Tai Chi practice location (24).

#### 2.2.2 Nature connectedness

Nature connectedness was assessed using the Connectedness to Nature Scale (CNS) developed by Mayer and Frantz (25), along with its validated Chinese version. The scale includes three dimensions: affective, cognitive, and experiential (26). The affective dimension reflects emotional experiences and emotional attachment to nature (27). The cognitive dimension reflects individuals’ understanding of the relationship between humans and nature. The experiential dimension reflects sensory perception and direct interaction with natural environments. All items were rated on a 5-point Likert scale (1 = strongly disagree, 5 = strongly agree). Reverse-coded items were recoded before analysis. Dimension scores were calculated as the sum or mean of corresponding items. Higher scores indicate stronger psychological connectedness with nature. In this study, the three dimension scores (affective, cognitive, and experiential) were used as continuous observed indicators and included in the Latent Profile Analysis (LPA) model (28). In this study, the internal consistency reliability of the Connectedness to Nature Scale was acceptable (Cronbach’s α = 0.81).

#### 2.2.3 Place attachment

Place attachment was assessed using the classic Place Attachment Scale developed by Williams and Vaske (29), which measures individuals’ psychological attachment to specific environments, in this case, urban park Tai Chi practice settings. The scale includes two core dimensions: place dependence and place identity (30). Place dependence reflects the functional reliance on the park environment, such as spatial conditions and convenience for Tai Chi practice (31). Place identity reflects emotional belonging and identity-based connection with the park. All items were rated on a 5-point Likert scale (1 = strongly disagree, 5 = strongly agree). Dimension scores were calculated as the sum or mean of corresponding items. Reverse-coded items were recoded before analysis. Higher scores indicate stronger place attachment. In this study, both place dependence and place identity were treated as continuous variables and included in subsequent analyses (32). In this study, the internal consistency reliability of the Place Attachment Scale was acceptable (Cronbach’s α = 0.83).

#### 2.2.4 Selection and preparation of indicators for latent profile analysis (LPA)

The indicators included in the LPA were selected based on the theoretical framework of this study and previous research on psychological relationships between individuals and environments. Specifically, nature connectedness and place attachment were considered the core psychological constructs reflecting older adults’ relationships with urban green spaces. Therefore, five continuous observed indicators were selected: the affective, cognitive, and experiential dimensions of nature connectedness, as well as place dependence and place identity.

Before conducting LPA, data screening procedures were performed. First, questionnaires with missing values or logically inconsistent responses were excluded during data cleaning. Second, descriptive statistics and distribution characteristics of the five indicators were examined to identify potential outliers and assess the suitability of the variables for continuous latent profile modeling. Third, all five indicators were standardized using z-score transformation (mean = 0, standard deviation = 1) to eliminate differences in measurement scales and ensure comparability across indicators (33).

Other behavioral variables (e.g., Tai Chi practice frequency, duration, and years of practice) were not included as LPA indicators because they were considered contextual characteristics rather than psychological classification variables. These variables were subsequently used to examine differences among latent profiles (34).

#### 2.2.5 Scoring procedures

All scales were scored in a consistent direction, with higher scores indicating stronger levels of the corresponding psychological constructs (35). Reverse-coded items were recoded prior to analysis. Dimension scores were calculated as the sum or mean of the corresponding items (36). Before conducting Latent Profile Analysis, the five continuous variables included in the model (i.e., the dimensions of nature connectedness and place attachment) were standardized using z-score transformation (37). This procedure ensured a mean of 0 and a standard deviation of 1 for each variable, thereby eliminating scale differences and ensuring comparability across indicators. Categorical variables were coded according to their original categories. Continuous variables were examined using descriptive statistics and tests of normality prior to analysis to meet the statistical assumptions required for latent profile analysis and subsequent variance analyses.

### 2.3 Statistical analysis

All analyses were performed using SPSS 26.0 and Mplus 8.3 software. The analytical procedure included four main steps: descriptive statistics, correlation analysis, latent profile analysis, and group difference testing (38).

First, descriptive statistics were conducted to summarize sample characteristics, including means, standard deviations, and frequencies. Pearson correlation analysis was used to examine the relationships among the dimensions of nature connectedness and place attachment, assessing linear associations between variables (39).

Second, Latent Profile Analysis (LPA) was performed to identify heterogeneous subgroups based on nature connectedness and place attachment. Five continuous indicators were included in the model: affective, cognitive, and experiential dimensions of nature connectedness, as well as place dependence and place identity (40). Prior to model estimation, the suitability of the selected indicators was evaluated through descriptive statistics and data screening procedures. No severe distributional problems or extreme outliers were identified. A series of models with 1 to 6 latent classes were estimated. Model fit was evaluated using multiple indices, including Akaike Information Criterion (AIC), Bayesian Information Criterion (BIC), sample-size adjusted BIC (aBIC), and entropy (41). Lower values of AIC, BIC, and aBIC indicate better model fit, while entropy values closer to 1 indicate higher classification accuracy (42). In addition, the Lo–Mendell–Rubin likelihood ratio test (LMR) and bootstrap likelihood ratio test (BLRT) were used to compare competing models. The optimal number of classes was determined by considering statistical fit indices, classification quality, theoretical interpretability, and practical relevance. After identifying the optimal model, latent profiles were labeled based on the score patterns across the five indicators, and the proportion of each class was calculated (43).

Finally, differences among latent profiles were examined using appropriate statistical methods according to the measurement characteristics of variables. One-way ANOVA was conducted to compare continuous variables across the three latent profiles, including dimensions of nature connectedness, dimensions of place attachment, and Tai Chi-related behavioral variables. When the assumption of homogeneity of variance was met, F-tests were used; otherwise, robust statistical methods were applied. Categorical sociodemographic variables were not included in the ANOVA analysis. All statistical tests were two-tailed, with a significance level set at *p* < 0.05 (44).

## 3 Results

### 3.1 Sample characteristics and descriptive statistics

A total of 462 urban older adults practicing Tai Chi were included in this study (Table 2). The age distribution was mainly concentrated in the 65–74-year range. The largest proportion was aged 65–69 years (36.6%), followed by those aged 70–74 years (31.4%). The gender distribution was relatively balanced, with a slightly higher proportion of females (53.2%). Regarding education level, most participants had a high school/technical secondary school education or below (77.3%). Monthly income was mainly concentrated in the 2000–3,999 RMB range (36.8%). Regarding the recruitment environment, 44.4% of participants were surveyed in comprehensive parks, 35.1% in community parks, and 20.6% in public squares, indicating that the sample covered diverse types of urban public spaces (Table 1). Overall, the sample reflected typical characteristics of urban community-dwelling older adults and showed a relatively high level of engagement in Tai Chi practice.

**Table 2:** Sociodemographic characteristics of the sample (*N* = 462).

Variable	Category	Frequency	Percentage (%)
Age	60–64 years	96	20.8
	65–69 years	169	36.6
	70–74 years	145	31.4
	75–80 years	52	11.3
Gender	Male	216	46.8
	Female	246	53.2
Education level	Primary school or below	55	11.9
	Junior high school	134	29.0
	High school/Technical secondary school	168	36.4
	College degree or above	105	22.7
Previous occupation	Worker/Farmer	171	37.0
	Enterprise staff	125	27.1
	Professional/Technical	101	21.9
	Managerial/Administrative	65	14.1
Monthly income	< 2000 RMB	161	34.8
	2000–3,999 RMB	170	36.8
	4,000–5,999 RMB	82	17.7
	≥ 6,000 RMB	49	10.6
Tai Chi practice frequency	3–4 times/week	97	21.0
	5 times/week	178	38.5
	6–7 times/week	187	40.5
Practice duration	≤ 40 min	125	27.1
	41–50 min	174	37.7
	51–60 min	127	27.5
	> 60 min	36	7.8
Years of practice	≤ 3 years	112	24.2
	4–6 years	149	32.3
	7–10 years	159	34.4
	> 10 years	42	9.1
Park type	Comprehensive park	205	44.4
	Community park	162	35.1
	Square	95	20.6

### 3.2 Correlation analysis between nature connectedness and place attachment

Pearson correlation analysis results are presented in Table 3 and Figure 2. The results showed that all dimensions of nature connectedness were significantly and positively correlated with those of place attachment (*p* < 0.01). Among the dimensions of nature connectedness, the correlations among affective, cognitive, and experiential dimensions ranged from 0.575 to 0.629. For place attachment, place dependence was strongly correlated with place identity (r = 0.630, *p* < 0.01). In addition, all dimensions of nature connectedness showed moderate to strong positive correlations with all dimensions of place attachment (r = 0.486–0.660), indicating a stable psychological association between the two constructs.

**Table 3:** Descriptive statistics and correlation matrix of core variables (*N* = 462).

Variable	M	SD	1	2	3	4	5
1. Affective dimension	15.91	3.98	1				
2. Cognitive dimension	15.70	3.88	0.575**	1			
3. Experiential dimension	12.47	3.60	0.629**	0.599**	1		
4. Place dependence	18.71	5.34	0.549**	0.486**	0.602**	1	
5. Place identity	17.84	5.45	0.660**	0.513**	0.622**	0.630**	1

*p < 0.05, **p < 0.01.

**Figure 2:**
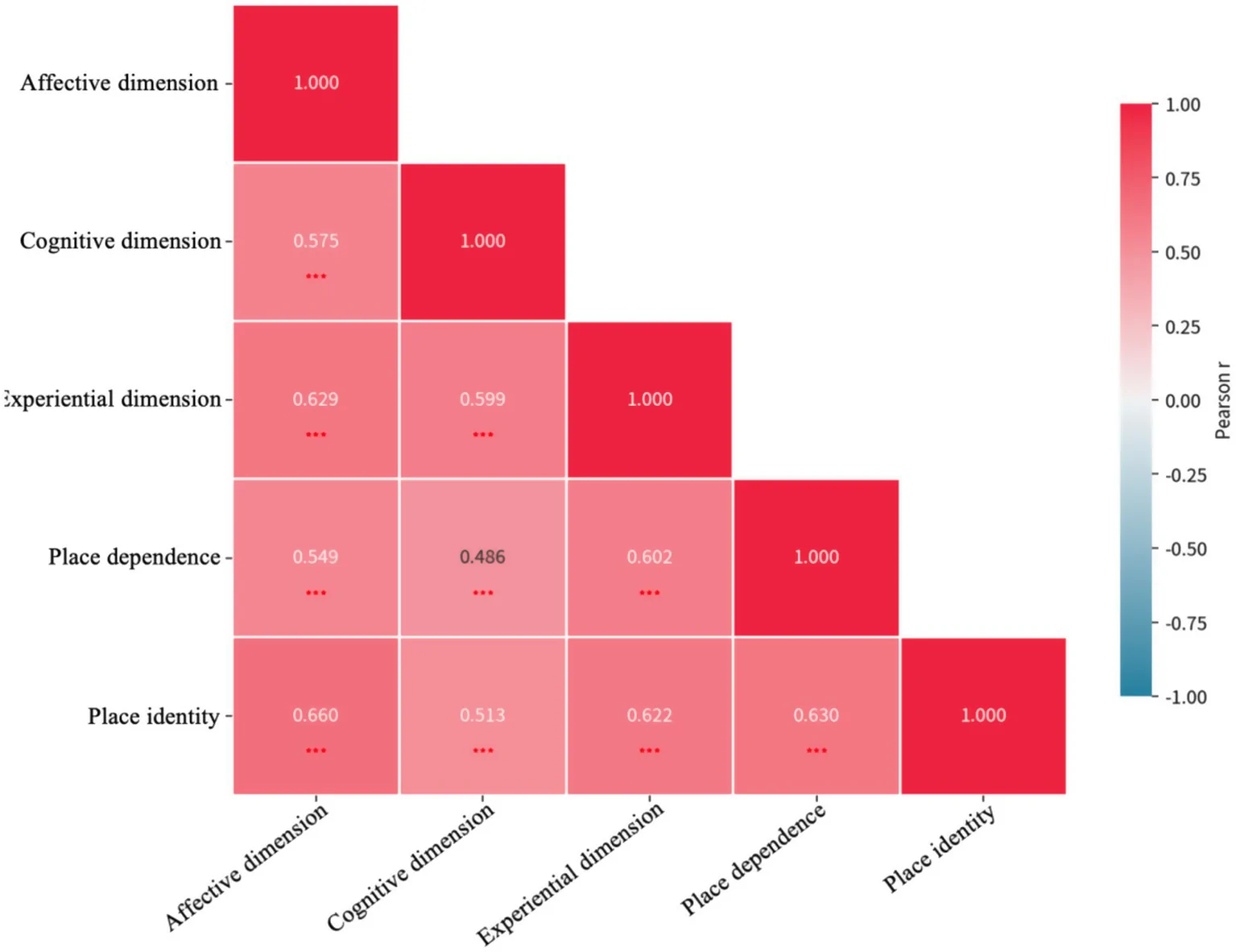
Heatmap of Pearson correlation coefficients among core variables.

### 3.3 Latent profile analysis results

#### 3.3.1 Selection of the optimal latent profile model

To identify the latent class structure of nature connectedness and place attachment among urban older adults practicing Tai Chi, a series of Latent Profile Analysis (LPA) models were estimated using five continuous indicators: affective, cognitive, and experiential dimensions of nature connectedness, as well as place dependence and place identity. Models with 1 to 6 classes were tested. Model fit indices, including Akaike Information Criterion (AIC), Bayesian Information Criterion (BIC), sample-size adjusted BIC (aBIC), entropy, and likelihood ratio tests (LMR and BLRT), are presented in Table 4.

**Table 4:** Model fit indices for latent profile analysis.

Class	AIC	BIC	aBIC	LMR(p)	BLRT(p)	Entropy	Class membership (n)
1	11758.704	11791.788	11766.399	—	—	—	462
2	10791.077	10861.381	10807.428	0.000	0.000	0.879	251, 211
3	10588.043	10695.568	10613.051	0.000	0.000	0.857	171, 146, 145
4	10523.424	10668.168	10557.088	0.082	0.000	0.821	141, 129, 126, 66
5	10478.605	10660.570	10520.926	0.214	0.000	0.802	123, 111, 107, 76, 45
6	10453.732	10672.917	10504.709	0.356	0.000	0.806	110, 102, 102, 56, 47, 45

AIC, Akaike Information Criterion; BIC, Bayesian Information Criterion; aBIC, sample-size adjusted Bayesian Information Criterion; LMR, Lo–Mendell–Rubin likelihood ratio test; BLRT, bootstrap likelihood ratio test.

Overall, AIC, BIC, and aBIC values decreased as the number of classes increased from 1 to 6, indicating improved model fit with additional classes. However, model selection should balance statistical fit with theoretical interpretability and avoid over-extraction, which may lead to unstable or fragmented class solutions.

Specifically, the two-class model showed a significant improvement over the one-class model, with substantially lower AIC, BIC, and aBIC values. The entropy value reached 0.879, and both the LMR and BLRT tests were statistically significant (*p* < 0.001), indicating that the two-class solution was superior to the single-class model.

The three-class model further improved model fit (AIC = 10588.043, BIC = 10695.568, aBIC = 10613.051). The entropy remained high (0.857), and both LMR and BLRT remained statistically significant (*p* < 0.001), suggesting that the three-class solution was significantly better than the two-class model.

For the four-class model, AIC, BIC, and aBIC continued to decrease slightly (AIC = 10523.424, BIC = 10668.168, aBIC = 10557.088), but the magnitude of improvement was reduced, indicating diminishing returns. In addition, the LMR test was no longer statistically significant (*p* = 0.082), suggesting no significant improvement compared with the three-class model. The smallest class size included only 66 individuals (14.3%), and increased overlap among classes reduced interpretability and classification stability.

For the five- and six-class models, LMR tests were also non-significant (*p* = 0.214 and *p* = 0.356, respectively), and entropy decreased to 0.802 and 0.806, indicating no improvement in classification accuracy. These results further supported the decision not to extract additional classes.

Considering model fit indices, likelihood ratio tests, classification quality, and theoretical interpretability, the three-class model was selected as the optimal solution. The selected three-class solution provided the most interpretable classification pattern, and additional classes did not produce theoretically meaningful subgroups representing divergent combinations of nature connectedness and place attachment. This model achieved a balance between statistical fit and parsimony. In addition, all classes had adequate sample sizes (*n* = 171, 146, and 145), indicating good model stability and practical interpretability.

#### 3.3.2 Characteristics of latent profiles

Based on the score patterns of nature connectedness and place attachment indicators, three distinct latent profiles were identified (Figure 3, Table 5). Although theoretically possible, profiles characterized by discrepant combinations of nature connectedness and place attachment (e.g., high connectedness–low attachment or low connectedness–high attachment) were not identified as independent latent classes in the present sample. This suggests that, among older adults practicing Tai Chi in urban green spaces, nature connectedness and place attachment tended to develop in a relatively coordinated manner rather than forming completely independent psychological patterns.

(1) High connectedness–High attachment type (C1, *n* = 154, 33.3%)

**Figure 3:**
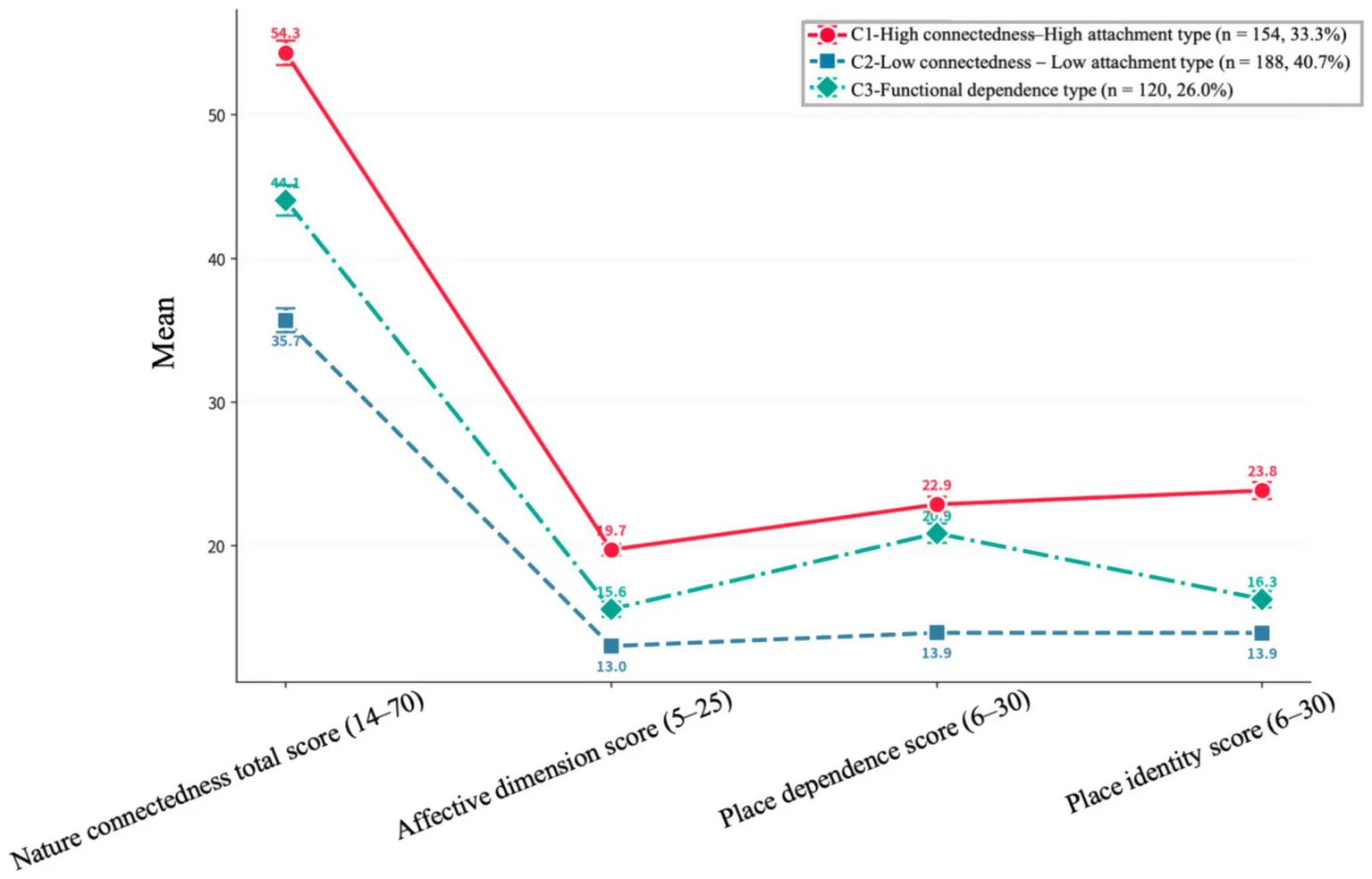
Latent profile plot of nature connectedness and place attachment among urban older adults practicing Tai Chi.

**Table 5:** Naming and characteristics of latent profiles of nature connectedness and place attachment.

Latent profile	Sample size *n* (%)	Level of nature connectedness	Level of place attachment
C1 High connectedness–High attachment type	154 (33.3%)	High	High
C2 Low connectedness–Low attachment type	188 (40.7%)	Low	Low
C3 Functional dependence type	120 (26.0%)	Moderate	Moderate

Individuals in this profile showed high levels across all dimensions of nature connectedness and place attachment. Specifically, they reported relatively high scores in the affective, cognitive, and experiential dimensions of nature connectedness, as well as in place dependence and place identity. This profile reflects a strong overall psychological connection to nature, along with stable functional dependence on and emotional identification with the Tai Chi practice setting. It represents a dual-high pattern of “nature connectedness–place attachment”.

(2) Low connectedness–Low attachment type (C2, *n* = 188, 40.7%)

Individuals in this profile showed consistently low levels across all dimensions of nature connectedness and place attachment. They reported low scores in the affective, cognitive, and experiential dimensions of nature connectedness, as well as in place dependence and place identity. This group demonstrated weak emotional engagement with nature and limited cognitive awareness of the human–nature relationship. They also showed low functional dependence on and emotional attachment to the practice environment, indicating the weakest overall psychological connection.

(3) Functional dependence type (C3, *n* = 120, 26.0%)

This profile showed a relatively balanced but distinct pattern rather than a polarized combination of high place attachment with low nature connectedness. Scores of nature connectedness were at a moderate level, and place identity was also moderate. However, place dependence was relatively higher compared with other dimensions. This pattern suggests that attachment to the practice environment is mainly driven by functional needs, such as spatial conditions and convenience for Tai Chi practice, rather than emotional identification or deeper nature experience. It reflects a function-oriented pattern of place relationship.

Overall, the proportions of the three profiles were relatively balanced (37.0, 31.6, and 31.4%, respectively), indicating clear heterogeneity in the psychological structure of nature connectedness and place attachment among urban older adults practicing Tai Chi. Each subgroup also represented a substantial proportion of the sample.

### 3.4 Differences in sociodemographic characteristics across latent profiles

Chi-square tests were conducted to examine differences in sociodemographic characteristics across latent profiles. The results showed significant differences among the three profiles in age, gender, education level, previous occupation, and monthly income (*p* < 0.05) (Table 6).

**Table 6:** Comparison of sociodemographic characteristics across three latent profiles.

Variable	Category	C1 (*n* = 154)	C2 (*n* = 188)	C3 (*n* = 120)	Total	*χ* ^2^	p
Age	60–64 years	60 (38.96)	28 (14.89)	8 (6.67)	96 (20.78)	83.712	<0.001**
	65–69 years	68 (44.16)	63 (33.51)	38 (31.67)	169 (36.58)		
	70–74 years	23 (14.94)	72 (38.30)	50 (41.67)	145 (31.39)		
	75–80 years	3 (1.95)	25 (13.30)	24 (20.00)	52 (11.26)		
Gender	Male	64 (41.56)	103 (54.79)	49 (40.83)	216 (46.75)	8.233	0.016*
	Female	90 (58.44)	85 (45.21)	71 (59.17)	246 (53.25)		
Education level	Primary school or below	9 (5.84)	34 (18.09)	12 (10.00)	55 (11.90)	33.508	<0.001**
	Junior high school	40 (25.97)	59 (31.38)	35 (29.17)	134 (29.00)		
	High school/Technical secondary school	50 (32.47)	72 (38.30)	46 (38.33)	168 (36.36)		
	College degree or above	55 (35.71)	23 (12.23)	27 (22.50)	105 (22.73)		
Previous occupation	Worker/Farmer	44 (28.57)	82 (43.62)	45 (37.50)	171 (37.01)	14.164	0.028*
	Enterprise staff	50 (32.47)	49 (26.06)	26 (21.67)	125 (27.06)		
	Professional/technical	38 (24.68)	38 (20.21)	25 (20.83)	101 (21.86)		
	Managerial/administrative	22 (14.29)	19 (10.11)	24 (20.00)	65 (14.07)		
Monthly income	<2000 RMB	40 (25.97)	90 (47.87)	31 (25.83)	161 (34.85)	25.866	<0.001**
	2000–3,999 RMB	61 (39.61)	60 (31.91)	49 (40.83)	170 (36.80)		
	4,000–5,999 RMB	33 (21.43)	26 (13.83)	23 (19.17)	82 (17.75)		
	≥6,000 RMB	20 (12.99)	12 (6.38)	17 (14.17)	49 (10.61)		

*p < 0.05, **p < 0.01; data are presented as frequency (percentage %).

In terms of age distribution, significant differences were observed among the three profiles (*χ*^2^ = 83.712, *p* < 0.001). The C1 group was mainly composed of individuals aged 60–69 years, with a relatively higher proportion in the 60–64-year group (38.96%). In contrast, both the C2 and C3 groups were more concentrated in the age group of 70 years and above. Notably, the C3 group had the highest proportion in the 75–80-year group (20.00%), indicating clear differences in age structure across profiles.

Regarding gender, significant differences were found among the profiles (*χ*^2^ = 8.233, *p* = 0.016). The C2 group had a relatively higher proportion of males (54.79%), while the C1 and C3 groups showed slightly higher proportions of females (58.44 and 59.17%, respectively).

For education level, significant group differences were observed (*χ*^2^ = 33.508, *p* < 0.001). The C1 group had the highest proportion of participants with college education or above (35.71%), whereas the C2 group had the highest proportion of primary education or below (18.09%). The C3 group showed an intermediate distribution.

Regarding previous occupation, significant differences were also identified (*χ*^2^ = 14.164, *p* = 0.028). The C2 group had a relatively higher proportion of workers/farmers (43.62%), while the C1 group showed a more diverse occupational distribution.

For monthly income, significant differences were observed across profiles (*χ*^2^ = 25.866, *p* < 0.001). The C2 group had a higher proportion of low-income participants (<2000 RMB, 47.87%), whereas the C1 group had a relatively higher proportion in the middle- and high-income categories.

Overall, the three latent profiles showed systematic differences in age structure, socioeconomic status, and other demographic characteristics.

### 3.5 Differences in psychological and behavioral variables across latent profiles

One-way analysis of variance (ANOVA) was conducted to examine differences in continuous psychological and behavioral variables across latent profiles. The results are presented in Table 7. Significant differences were observed among the three latent profiles in all dimensions of nature connectedness and place attachment (all *p* < 0.001), including the affective, cognitive, and experiential dimensions, as well as place dependence, place identity, and total place attachment.

**Table 7:** Comparison of continuous variables across latent profiles.

Variable	C1 (*n* = 154)	C2 (*n* = 188)	C3 (*n* = 120)	*F*	*p*
Affective dimension	19.71 ± 2.62	13.01 ± 2.77	15.57 ± 2.90	251.918	<0.001***
Cognitive dimension	18.66 ± 2.96	13.06 ± 3.04	16.02 ± 3.17	143.896	<0.001***
Experiential dimension	15.95 ± 2.44	9.63 ± 2.24	12.46 ± 2.48	300.900	<0.001***
Place dependence	22.87 ± 3.32	13.93 ± 3.52	20.87 ± 3.71	306.035	<0.001***
Place identity	23.84 ± 3.83	13.93 ± 2.84	16.27 ± 3.19	402.578	<0.001***
Total place attachment	46.71 ± 5.86	27.86 ± 5.00	37.14 ± 5.56	508.715	<0.001***
Total nature connectedness	54.32 ± 5.49	35.69 ± 5.81	44.06 ± 5.89	448.605	<0.001***
Practice frequency	5.23 ± 0.99	5.39 ± 1.04	5.24 ± 0.97	1.266	0.283
Practice duration	48.67 ± 8.65	50.37 ± 10.14	46.92 ± 10.13	4.751	0.009**
Years of practice	5.69 ± 2.96	5.64 ± 3.22	6.22 ± 3.26	1.394	0.249
Distance to park	18.47 ± 6.42	18.88 ± 7.09	18.69 ± 6.40	0.159	0.853

*p < 0.05, **p < 0.01, ***p < 0.001; values are presented as mean ± standard deviation (M ± SD).

For nature connectedness, the C1 group showed significantly higher scores in the affective dimension (19.71 ± 2.62), cognitive dimension (18.66 ± 2.96), and experiential dimension (15.95 ± 2.44) compared with the C2 group (13.01 ± 2.77, 13.06 ± 3.04, and 9.63 ± 2.24, respectively), while the C3 group remained at intermediate levels (*p* < 0.001). The overall nature connectedness score also showed significant differences among groups (*F* = 448.605, *p* < 0.001).

For place attachment, the C1 group had the highest scores in place dependence (22.87 ± 3.32) and place identity (23.84 ± 3.83), whereas the C2 group showed the lowest values (13.93 ± 3.52 and 13.93 ± 2.84, respectively), with the C3 group remaining at intermediate levels. The total place attachment score also differed significantly among the three profiles (*F* = 508.715, *p* < 0.001).

Among behavioral variables, only Tai Chi practice duration showed a significant difference across latent profiles (*F* = 4.751, *p* = 0.009), with the C2 group reporting slightly longer duration (50.37 ± 10.14 min) compared with the C1 and C3 groups (48.67 ± 8.65 and 46.92 ± 10.13 min, respectively). Regarding behavioral characteristics, most variables did not differ significantly among the three latent profiles, except for practice duration (*F* = 4.751, *p* = 0.009).

## 4 Discussion

### 4.1 Main findings and key contributions

Based on a person-centered approach (Latent Profile Analysis), this study identified three distinct profiles of urban older adults practicing Tai Chi: the High connectedness–High attachment type, the Low connectedness–Low attachment type, and the Functional dependence type (45). The results indicated that these profiles showed clear stratification in psychological variables. Interestingly, the expected contrasting patterns of high nature connectedness with low place attachment, or low nature connectedness with high place attachment, did not emerge as independent profiles. One possible explanation is that Tai Chi practice in urban green spaces involves repeated physical presence, sensory interaction with nature, and long-term use of specific places. These processes may simultaneously reinforce both individuals’ emotional connection with nature and their attachment to the practice environment. Therefore, nature connectedness and place attachment may exhibit a relatively coupled pattern within this specific population. In contrast, differences in most behavioral variables were limited, with only practice duration showing a slight difference across profiles. These findings suggest that psychological experiences related to nature and place may be more closely associated with perceived environmental adaptation and psychological resources than behavioral frequency alone (46).

The main contribution of this study lies in integrating nature connectedness and place attachment into a unified analytical framework. By applying Latent Profile Analysis, this study further revealed intra-group heterogeneity, providing new person-centered evidence for understanding the relationship between older adults and urban green spaces (47).

### 4.2 Potential contextual mechanisms linking nature connectedness, place attachment, and tai chi practice environment

The results of this study showed a stable positive association between nature connectedness and place attachment, which further formed a clear latent profile structure. This suggests that psychological experiences of older adults in urban parks may reflect relatively consistent patterns of environmental cognition and emotional response (48).

Within the context of Tai Chi practice, the activity may provide a favorable setting in which repeated interaction with natural environments, stable spatial use, and social engagement could be associated with stronger nature connectedness and place attachment. However, because this study did not include a non-Tai Chi comparison group, whether these psychological patterns are specific to Tai Chi practice or reflect broader characteristics of older adults engaging in urban green spaces remains unclear. As a low-intensity and slow-paced mind–body exercise, Tai Chi is typically practiced in urban green spaces and may influence psychological processes through several pathways. First, Tai Chi practice requires sustained attention and body awareness, which may be related to increased sensitivity to surrounding environmental cues and enhanced perception of natural elements. Second, Tai Chi is usually practiced in fixed locations and at regular time intervals over long periods. This stable spatial usage pattern may be associated with increased familiarity with and repeated use of specific places. Third, the group-based nature of morning exercise may enhance social interaction and emotional experience, which may be reflected in more positive evaluations of the environment (49).

Therefore, Tai Chi practice may be associated with higher levels of nature connectedness and place attachment through several possible pathways, stable spatial usage, and strengthened social–emotional experience (50).

### 4.3 Sociodemographic differences across latent profiles and their implications

Chi-square analyses indicated significant differences in age distribution, education level, income category, and occupational background across latent profiles. The High connectedness–High attachment group (C1) was generally characterized by younger age, higher education, and higher income levels, whereas the Low connectedness–Low attachment group (C2) was more concentrated among older individuals with lower education and lower income levels. This distribution suggests that socioeconomic resources may, to some extent, influence individuals’ cognitive capacity for environmental interpretation and depth of spatial engagement (51).

From a life-course perspective, higher education may be associated with better environmental cognition and health-related information processing ability, thereby facilitating a better understanding and more positive experience of natural environments. Similarly, higher income may be related to greater access to and utilization of higher-quality green spaces. The combined effect of These factors may be associated with stronger patterns of nature connectedness and place attachment among higher-resource groups (52).

Notably, the Functional dependence group (C3) showed a more complex pattern in sociodemographic characteristics. For example, although this group was generally older, it still demonstrated moderate levels of place dependence. This may suggest a transitional pattern in which individuals rely on green spaces primarily for functional purposes while gradually developing stronger emotional attachment. This profile provides important implications for stratified and targeted health promotion strategies (53).

### 4.4 Limited behavioral differences and the potential “psychological precedence” mechanism

An important finding of this study is that most behavioral variables, such as practice frequency, years of practice, and distance to parks, did not differ significantly across latent profiles, with the exception of practice duration. This suggests that behavioral indicators alone may be insufficient to distinguish psychological experience patterns related to urban green spaces among older adults (54).

These findings may indicate that psychological profiles provide additional information beyond behavioral indicators, indicating that individuals with similar behavioral patterns (e.g., frequent Tai Chi participation) may still exhibit substantially different internal psychological structures. In other words, behavioral similarity may not necessarily reflect psychological homogeneity (55).

From a health promotion perspective, this result is meaningful. Traditional classification approaches based mainly on behavioral frequency may underestimate potential heterogeneity within populations. In contrast, psychological profiles based on nature connectedness and place attachment may provide a more precise representation of individuals’ relationships with their environments and offer a more refined basis for targeted intervention design (56).

### 4.5 Implications for public health and urban green space planning

Based on the latent profile analysis results, this study identified significant heterogeneity in nature connectedness and place attachment among urban older adults practicing Tai Chi, revealing three distinct profiles: the High connectedness–High attachment type, the Low connectedness–Low attachment type, and the Functional dependence type. These findings suggest that health promotion strategies in urban green spaces may benefit from moving beyond uniform population-based approaches and adopt stratified interventions tailored to different psychological profiles (57).

For individuals in the Low connectedness–Low attachment group, both nature connectedness and place identity were relatively weak, and this group was more likely to be concentrated among individuals with lower socioeconomic resources. For this subgroup, urban park interventions may benefit from prioritizing initial environmental exposure and participation opportunities. For example, low-threshold group-based Tai Chi sessions could be organized in community parks, and structured morning exercise programs at fixed time slots may be considered to increase engagement with green spaces. Such approaches may gradually strengthen environmental familiarity and basic psychological connection (58).

For individuals in the Functional dependence group, a pattern of relatively high place dependence but low place identity was observed, suggesting a predominantly function-oriented use of green spaces with limited emotional attachment. For this subgroup, intervention strategies may consider shifting from functional accessibility toward enhancing emotional experience. This may include improving the visual quality of natural landscapes, introducing seasonal vegetation diversity, and incorporating culturally meaningful Tai Chi-related elements into park design, thereby strengthening place identity and emotional bonding (59).

For individuals in the High connectedness–High attachment group, relatively stable and strong psychological connections with both nature and place were observed. This group may serve as a core population maintaining the health benefits of urban green spaces. For this subgroup, planning strategies may focus on maintaining long-term positive experiences, such as ensuring spatial stability of practice areas, minimizing functional conflicts in park use, and providing supportive social interaction facilities to sustain continuous engagement and psychological benefits (60).

Overall, these findings highlight the importance of shifting urban green space planning and health promotion strategies from a uniform design model toward a stratified, profile-based intervention approach. By tailoring environmental and behavioral interventions according to heterogeneous psychological characteristics, the overall effectiveness of urban green spaces in promoting active aging and mental well-being may be enhanced (61).

## 5 Limitations and future perspectives

This study has several limitations. First, the sample selection criteria may have introduced selection bias. Participants were required to engage in regular Tai Chi practice in urban green spaces, which means that the sample mainly represented older adults with relatively stable and frequent Tai Chi participation rather than all older adults who practice Tai Chi occasionally. Therefore, the identified latent profiles may not fully capture the psychological patterns of less active practitioners or older adults with different exercise habits. Future studies should include participants with broader levels of Tai Chi involvement to improve the generalizability of the findings. Second, the sample was drawn from older adults practicing Tai Chi in urban parks in Xuzhou, Jiangsu Province, China. This regional and activity-specific sampling may limit the generalizability of the findings to other populations, regions, or non–Tai Chi practitioners. Third, the study relied mainly on self-reported questionnaires to assess psychological constructs, which may introduce subjective bias. In addition, objective environmental indicators (e.g., green space quality and air quality) and objective physical activity measures were not included. Fourth, although the sample was recruited from multiple types of urban public spaces and different time periods were considered, the use of convenience sampling may limit the generalizability of the findings. Older adults who regularly practice Tai Chi in public spaces may differ from those who exercise less frequently or use private fitness facilities. Therefore, future studies should consider probability-based sampling designs or larger multicenter samples to improve population representativeness.

In addition, although all participants were currently urban residents, some had previous agricultural occupational backgrounds. Previous rural living experiences may influence individuals’ relationships with natural environments and may therefore act as a potential confounding factor when interpreting nature connectedness. Future studies should further consider residential history and urban–rural migration experiences.

Future research may address these limitations in several ways. Longitudinal designs are recommended to explore dynamic changes in nature connectedness and place attachment during long-term Tai Chi participation. In addition, future studies could incorporate health outcomes such as mental health, cognitive function, and quality of life to examine differences in health trajectories across latent profiles. Moreover, combining objective environmental data and behavioral tracking could provide a more comprehensive understanding of the dynamic interactions among person, environment, and behavior. Finally, intervention studies based on profile-specific health promotion strategies are needed to enhance the application of urban green spaces and traditional mind–body exercises in promoting active aging.

## 6 Conclusion

This study adopted a person-centered approach using Latent Profile Analysis to identify heterogeneity in nature connectedness and place attachment among urban older adults practicing Tai Chi. Three distinct latent profiles were identified: the High connectedness–High attachment type, the Low connectedness–Low attachment type, and the Functional dependence type.

The results indicated clear stratification across psychological characteristics, while differences in most behavioral variables were relatively limited. This suggests that variations in older adults’ experiences of urban green spaces are primarily reflected at the psychological level rather than in behavioral frequency.

In addition, Tai Chi practice may be associated with the formation of nature connectedness and place attachment, highlighting its potential role in facilitating long-term engagement and psychological connection with urban green spaces among older adults.

Overall, these findings provide new person-centered evidence for understanding the complex relationship between older adults and green spaces. They also offer theoretical implications for health promotion strategies based on traditional mind–body practices and for optimizing urban public space design in the context of active aging.

## Data Availability

The original contributions presented in the study are included in the article/supplementary material, further inquiries can be directed to the corresponding author.
